# Supplementary material for: Potential and challenges for sustainable progress in human longevity
Source: Nat Commun. 2026 Jan 24;17:996. doi: 10.1038/s41467-026-68828-z (PMC12847798; doi:10.1038/s41467-026-68828-z)
Supplement: Supplementary file 1 — Supplementary Information [file 41467_2026_68828_MOESM1_ESM.pdf]

Supplementary Information for: Potential and challenges for sustainable progress in human longevity

Florian Bonnet[1], Ina Alliger[2], Carlo-Giovanni Camarda[1], Sebastian Klüsener[2,3,4], France Meslé[1], Michael Muhlichen[2], Josselin Thuilliez[5], and Pavel Grigoriev*[2]

[1] *French Institute for Demographic Studies (Ined), Aubervilliers, France*
[2] *Federal Institute for Population Research (BiB), Wiesbaden, Germany*
[3] *Institute of Sociology and Social Psychology (ISS), University of Cologne, Cologne, Germany*
[4] *Vytautas Magnus University, Kaunas, Lithuania.*
[5] *French National Centre for Scientific Research (CNRS), UMR 6211, Rennes, France*

* Corresponding author: Pavel Grigoriev, email: pavel.grigoriev@bib.bund.de

Supplementary Figure 1 presents the average annual gains in male (left panels) and female (right panels) life expectancy at birth for the periods 1992-2004 and 2005-2019. It complements the right panels of Figures 4 and 5.

Supplementary Figure 2 presents the change in the pace of 20q55 decrease against the change in the pace of life expectancy gains between 1992 and 2019 across 450 regions of Western Europe.

Supplementary Figure 3 presents the same outcomes as Figure 6 for the probability of dying between ages 35 and 54 (20q35).

Supplementary Figure 4 presents the same outcomes as Figure 6 for the probability of dying between ages 75 and 84 (10q75).

Supplementary Figure 5 illustrates our methods for 10 French and Dutch regions by presenting female life expectancy at birth derived from the raw data and from our model (with 95% confidence interval).

Supplementary Table 1 presents the same outcomes as Table 1 but for the periods 1992-2004 and 2005-2019.

Supplementary Table 2 presents the same outcomes as Table 2 but for the periods 1992-2004 and 2005-2019.

Supplementary Table 3 presents the raw data used and the minor adjustments made.

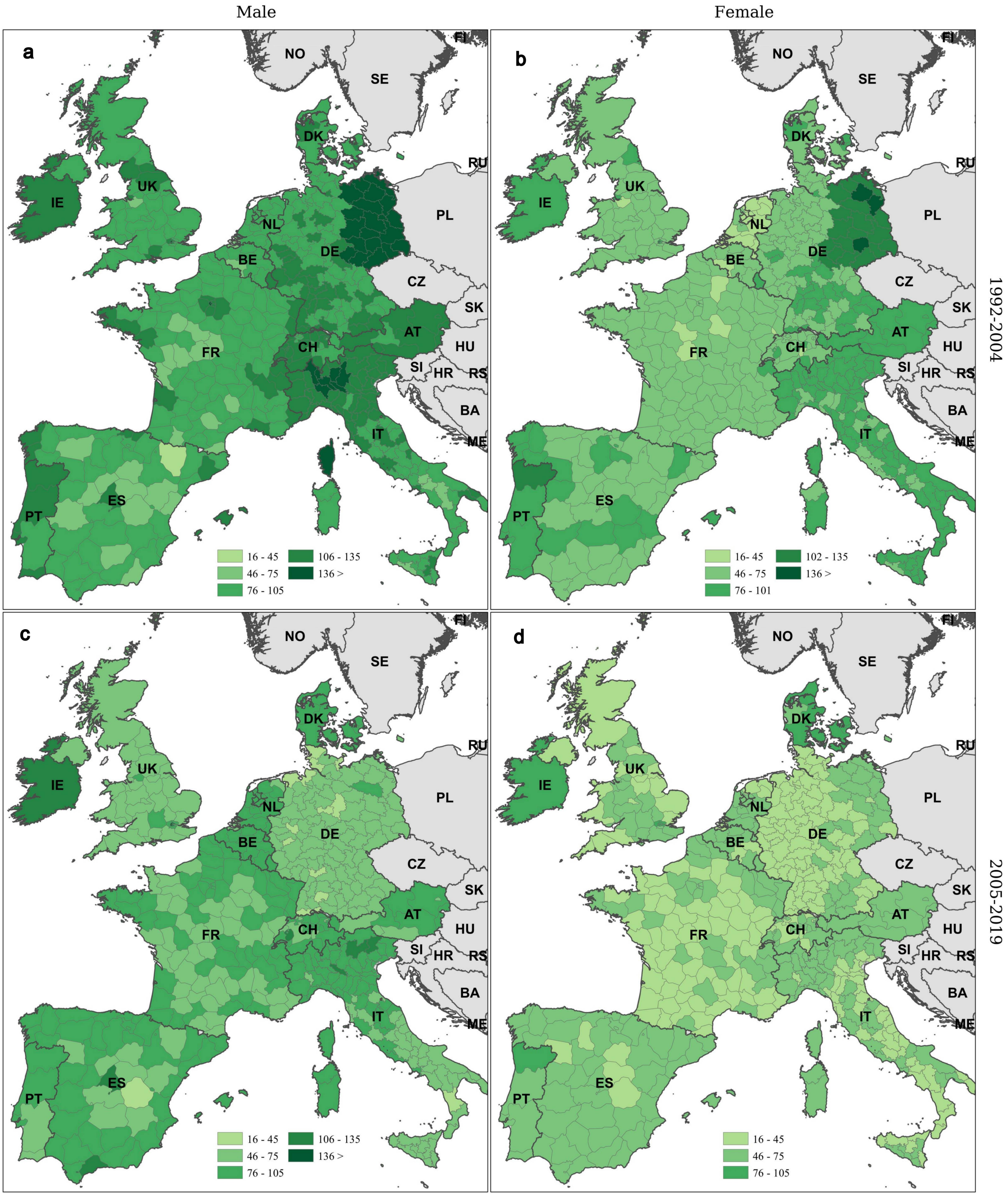

Supplementary Figure 1. Average annual gains in life expectancy (e_0) for males and female, in 1992–2004 and 2005-2019.

Average annual gains in e_0 are shown for males and females across the four panels. Panels a and c provide the values for males, panels b and d provide the values for females. For visual simplicity, the island regions of *Las Palmas* and *Tenerife* (ES) and the *Azores* and *Madeira* (PT) are not shown in maps in the paper. Source data are provided as a Source Data file.

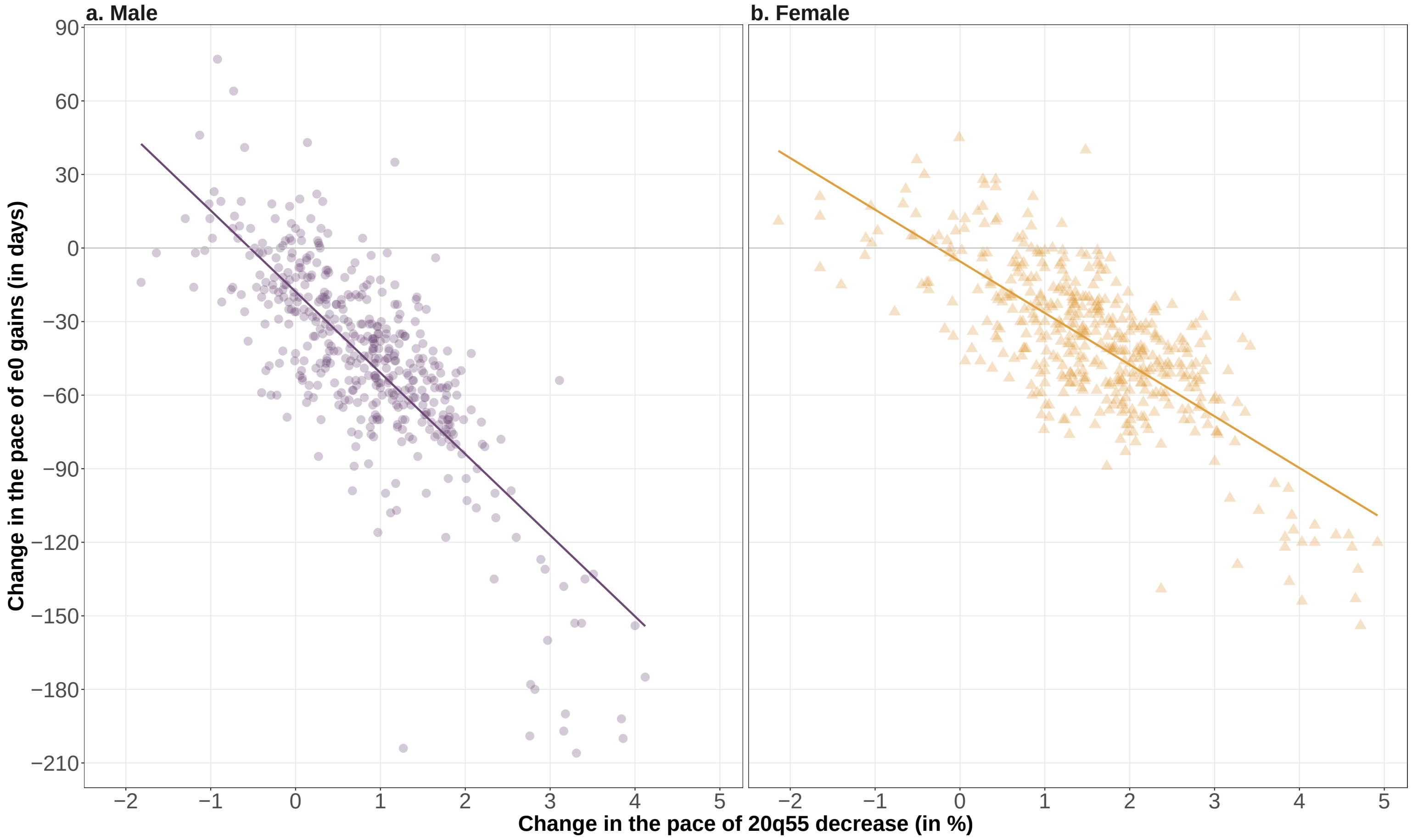

Supplementary Figure 2. Relationship between changes in the pace of decline in 20q55 (in %) and improvements in life expectancy (e_0) between 1992–1993 and 2018–2019, Western Europe.

The relationship between changes in the pace of decline in mortality between ages 55 and 74 (20q55) and changes in the pace of e_0 gains is shown for Western European regions. Panel a depicts the relationship for males, with each point representing a region and plotting the difference in the annual percentage change in 20q55 between 2018–2019 and 1992–1993 on the x-axis against the corresponding difference in annual gains in e_0 on the y-axis. Positive values on the x-axis indicate regions where the pace of mortality improvement has slowed between the early 1990s and the late 2010s, while negative values on the y-axis indicate regions where the pace of e_0 gains has slowed over the same period. Panel b depicts the same relationship for females. Source data are provided as a Source Data file.

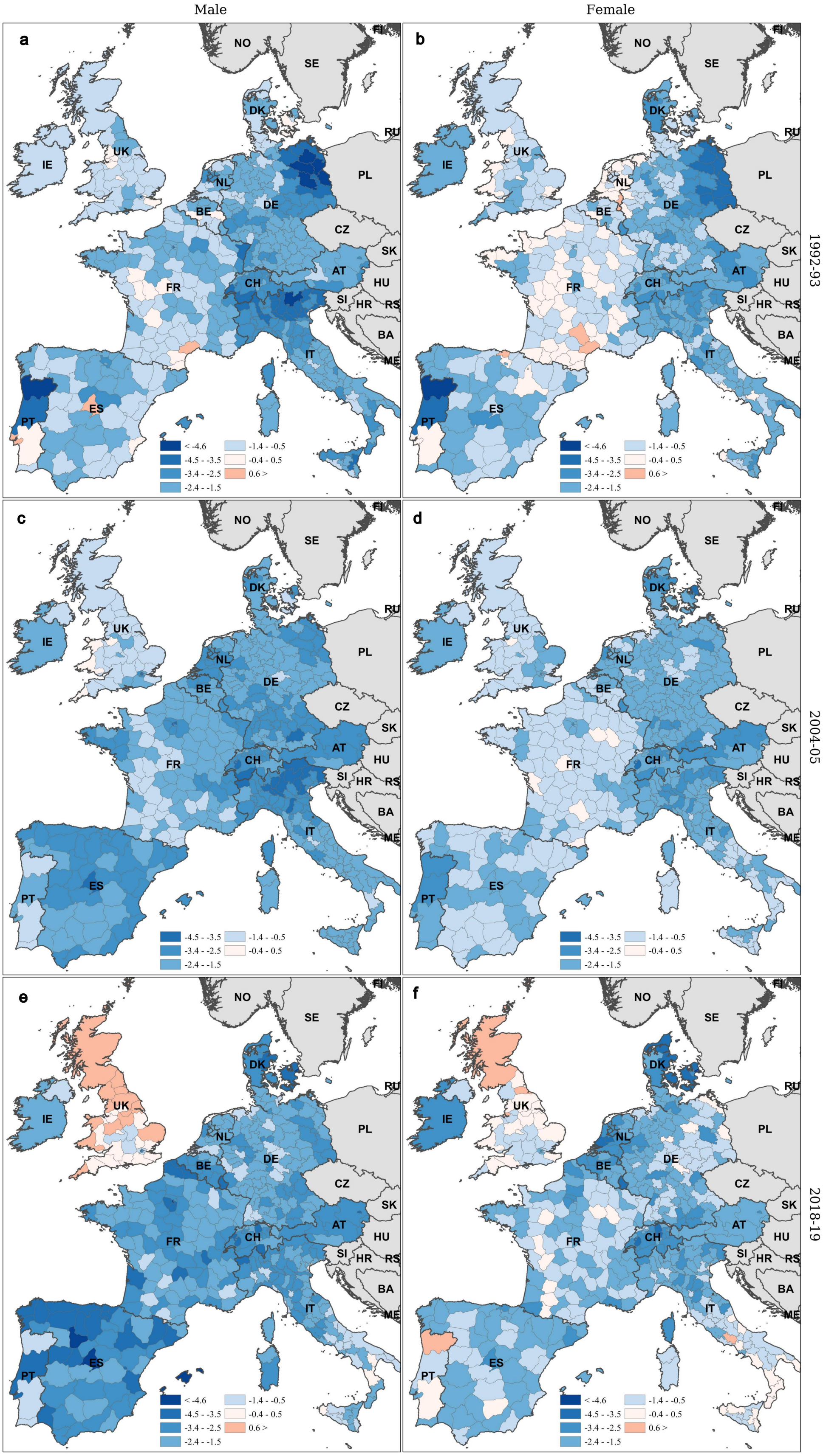

Supplementary Figure 3. Annual percentage change in the probability of dying between ages 35 and 54 (20q35) across 450 Western European regions in 1992–1993, 2004–2005, and 2018–2019. Annual percentage changes in the probability of dying between ages 35 and 54 are displayed across the six panels for males and females. Panels a, c and e provide the values for males, panels b, d and f provide the values for females. See Table 2 for more information about the variable. Source data are provided as a Source Data file.

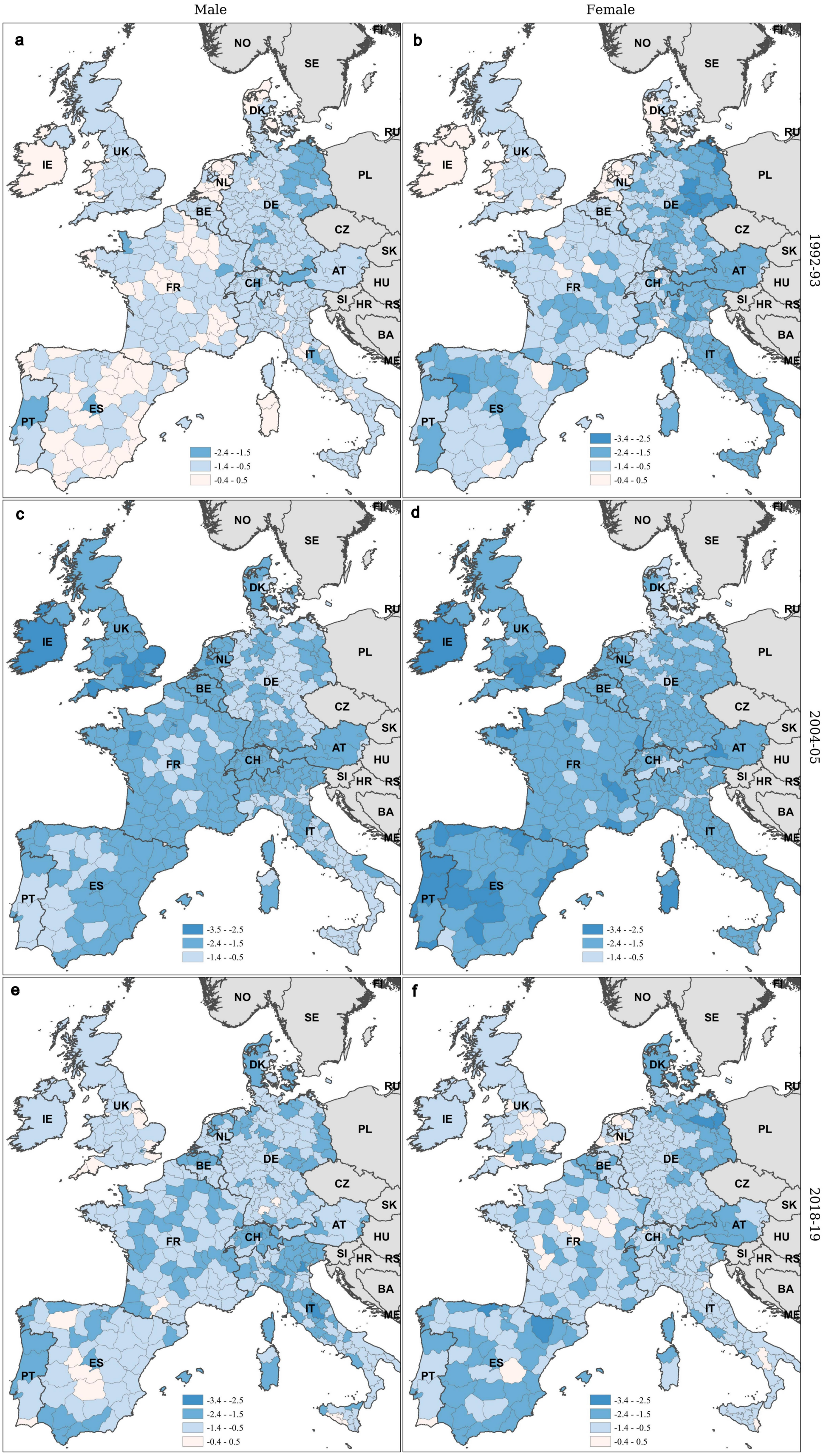

Supplementary Figure 4. Annual percentage change in the probability of dying between ages 75 and 84 (10q75) across 450 Western European regions; 1992–1993, 2004–2005, and 2018–2019. Annual percentage changes in the probability of dying between ages 75 and 84 are displayed across the six panels for males and females. Panels a, c and e provide the values for males, panels b, d and f provide the values for females. See Table 2 for more information about the variable. Source data are provided as a Source Data file.

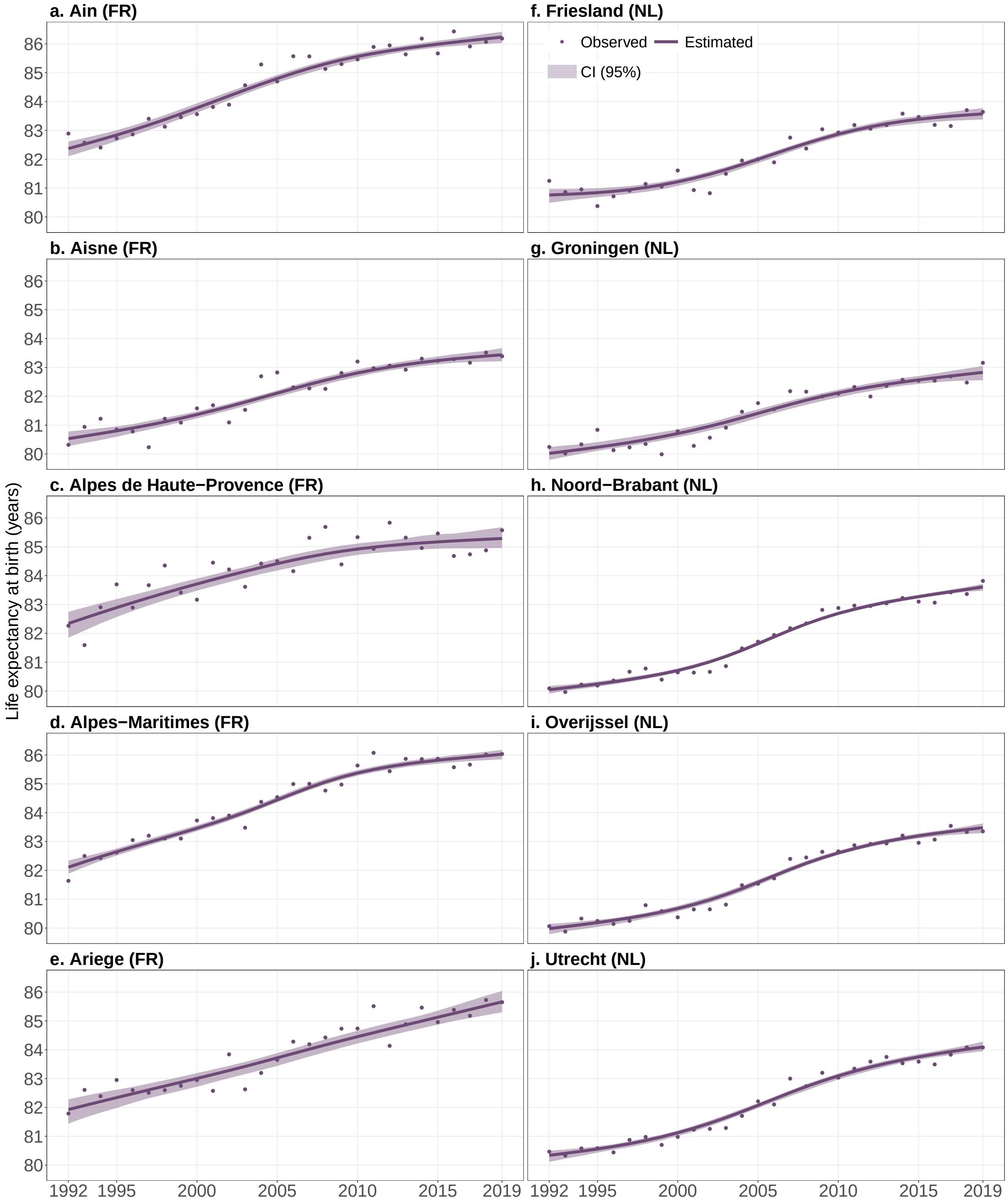

Supplementary Figure 5. Life expectancy at birth (e_0) for females, 1992–2019, across 5 regions in France and 5 regions in the Netherlands.
e_0 for females is shown across ten panels for selected regions in France and the Netherlands over the period 1992–2019. In all panels, points represent observed values directly from the data, while lines and shaded bands correspond to model-based estimates with 95% confidence intervals. Panels a to e depict trends for the five French regions, and panels f to j depict trends for the five Dutch regions. Source data are provided as a Source Data file.

Supplementary Table 1. Annual gains in life expectancy (in days) in vanguard and laggard regions of Western Europe for 1992–2019, 1992-2004 and 2005-2019.

	Male			Female		
	**1992-2019**	1992-04	2005-19	**1992-2019**	1992-04	2005-19
Maximum	**82**	69	94	**49**	52	46
Vanguard	**89**	94	83	**57**	63	51
Mean	**89**	103	75	**60**	72	48
Laggard	**97**	124	72	**69**	89	50
Minimum	**111**	104	117	**76**	91	62

Values for vanguard and laggard regions are based on the unweighted life expectancy at birth of regions in each group. Values for the mean are computed from the unweighted life expectancy at birth across the 450 European regions in the panel. Values in bold are for the whole period. Source data are provided as a Source Data file.

Supplementary Table 2. Average annual change (per cent) in the probability of dying by age range across 450 Western European regions, 1992–2019.

Prob. of dying between	Male			Female		
	**1992-2019**	1992-04	2005-19	**1992-2019**	1992-04	2005-19
35 and 54 (20q35)	**-2.3**	-2.1	-2.4	**-1.7**	-1.6	-1.8
55 and 74 (20q55)	**-1.8**	-2.1	-1.5	**-1.5**	-2.1	-1.0
75 and 84 (10q75)	**-1.4**	-1.3	-1.5	**-1.6**	-1.6	-1.5

The probability of dying between ages a_1 and a_2 is computed as the number of deaths between these ages divided by the number of individuals alive at age a_1 in the lifetable. Values in bold are for the whole period. Source data are provided as a Source Data file.

Supplementary Table 3. Regional division, sources, data information, and adjustments by country

**Country**	**Spatial units**	**Source**	**Age classification**	**Adjustments**
Austria	9 NUTS-2 units	Statistics Austria<br>Eurostat	Deaths: 0-4,5-9, . . . ,95+<br>Pop: 0,1, . . . ,100+	Upper age limit for pop. data until 2001: 95+
Belgium	11 NUTS-2 units	Belgian Statistical Office	0,1, . . . ,95+	
Denmark	11 NUTS-3 units	Statistics Denmark	Deaths:0,1, . . . ,99+<br>Pop: 0,1, . . . ,120+	Harmonized to apply current division (*)
France	95 NUTS-3 units	INSEE	0,1, . . . ,95+	Non-European areas excluded, Corsica merged
Germany	96 ROR	Stat. Offices of the<br>German Länder	Deaths: 0,1-4,5-9, . . . ,90+<br>Pop: 0,1, . . . ,90+	Harmonized to apply current territorial division (**)
Ireland	1 NUTS-0 unit	HMD<br>Eurostat	Deaths: 0,1, . . . ,110+<br>Pop: 0,1, . . . ,110+	Upper age limit for pop. and death counts 2021: 100+
Italy	92 NUTS-3 units	ISTAT	Deaths: 0-4,5-9, . . . ,95+<br>Pop: 0,1, . . . ,100+	Harmonized to maintain a consistent time series (***)
Luxembourg	1 NUTS-3 unit	HMD	Deaths: 0,1, . . . ,110+<br>Pop: 0,1, . . . ,95+	
Netherlands	12 NUTS-2 units	Stat. Netherlands	Deaths: 0,1-4,5-9, . . . ,95+<br>Pop: 0,1, . . . ,105+	Upper age limit for pop. data until 2015: 95+
Portugal	7 NUTS-2 units	Eurostat	0,1, . . . ,100+	
Spain	50 NUTS-3 units	Nat. Stat. Institute	0,1, . . . ,100+	Upper age limit for pop. data until 2001: 85+
Switzerland	26 NUTS-3 units	Fed. Stat. Office	Deaths: 0,1, . . . ,99+<br>Pop: 0,1, . . . ,100+	
UK	37 NUTS units	ONS (EW)<br><br>NISRA (North. I.)<br>NRS (Scot.)	EW<br>Deaths: 0,1-4,5-9, . . . ,95+<br>Pop: 0,1, ...,90+<br><br>North. I. & Scot.<br>Deaths: 0,1, ...,110+<br>Pop: 0,1, ..., 90+	Upper age limit for pop. data for EW until 2001: 85+<br>NUTS-2 level data for EW (aggregated from LAU)<br><br>NUTS-0 level data for North. I. and Scot.<br>due to data quality issues

(*) Due to a municipality reform in 2006, the municipality of Mariager (LAU code *DK7019*) was divided between NUTS3 regions *DK050* and *DK041*. Mariager was added to *DK050* (North Jutland).

(**) Harmonized to apply territorial division as of December 2022 to the whole study period and to eliminate the census 2011 break; 400 NUTS-3 units (‘Kreise) aggregated to 96 ROR units according to the classification of BBSR (2017).

(***) We merged the following regions: (1) Biella + Vercelli, (2) Novara + Verbano, (3) Como + Lecco, (4) Milano + Lodi + Monza + Brianza, (5) Rimini + Forli-Cesena, (6) Firenze + Prato, (7) Cagliari + Medio Campidano + Carbonia-Iglesias + Ogliastra + Oristano + Nuoro, (8) Sassari + Olbia-Tempio, (9) Foggia + Bari + Barletta, (10) Fermo + Ascoli-Piceno, (11) Crotone + Vibo Valentia + Cantanzaro.
